# Pilot study on the applicability of boron‐doped diamond electrodes for tooth whitening

**DOI:** 10.1002/cre2.586

**Published:** 2022-05-11

**Authors:** Virgilia Klär, Victor Palarie, Andreas Burkovski, Matthias Karl, Tanja Grobecker‐Karl

**Affiliations:** ^1^ Department of Prosthodontics Saarland University Homburg/Saar Germany; ^2^ Laboratory of Tissue Engineering and Cellular Cultures State University of Medicine and Pharmacy “N. Testemitanu” Chisinau Moldova; ^3^ Microbiology Division, Department of Biology University of Erlangen‐Nürnberg Erlangen Germany

**Keywords:** boron‐doped diamond electrodes, reactive oxygen species, shade selection, tooth whitening

## Abstract

**Objectives:**

While various approaches are available for tooth whitening, the basic concept employs the use of peroxides in the form of gels, which are applied to tooth surfaces. Previous studies have shown that reactive oxygen species acting as potent disinfectants can be produced using boron‐doped diamond (BDD) electrodes for the electrolysis of water. With these electrodes being applicable, for example, for endodontic treatment, it was the goal of this pilot study to use such electrodes for tooth whitening.

**Material and Methods:**

Two groups (*n* = 10) of intact clinical crowns were obtained by horizontally cutting off roots of extracted human teeth. The crowns were either bleached by applying a commercially available agent based on 40% hydrogen peroxide or were immersed in saline undergoing electrolysis with BDD electrodes. Whitening of specimens was judged on standardized photographs by examiners with three different levels of experience. Statistical analysis was based on Gwet's AC2 coefficient with quadratic weights, Shapiro–Wilk tests, and two‐way analysis of variance of aligned rank transformed data (level of significance set at *α* = .05).

**Results:**

Levels of reliability ranging from fair to substantial were recorded for single persons while the level of reliability ranged between fair and moderate for groups of raters. The level of experience had no significant effect on the ratings (*p* = .2500). The bleaching method had a significant effect on ratings (*p* = .0005) with BDD electrodes showing less effect.

**Conclusions:**

Bleaching by applying BDD electrodes was possible, but was not as effective as the use of commercially available in‐office whitening gel. A potential explanation may be seen in different concentrations of reactive oxygen species.

## 1 INTRODUCTION

Tooth whitening has been described as one of the most noteworthy advancements in esthetic dentistry (Blatz et al., 2019) and has become one of the most requested dental treatments (Rodríguez‐Martínez, 2019). Several approaches (Carey, 2014) with respect to materials and application modes such as in‐office bleaching, home bleaching (da Rosa et al., 2020) and internal bleaching (Frank et al., 2021) are available (Epple et al., 2019).

Most whitening systems use varying concentrations (Maran et al., 2020) of hydrogen peroxide as the active ingredient, which is either applied directly or produced in a chemical reaction from carbamide peroxide (Carey, 2014; Frank et al., 2021; Rodríguez‐Martínez et al., 2019). The mode of action consists of the oxidation of organic chromophores (Epple et al., 2019) and the effectiveness seems to depend on factors such as concentration and pH value of the whitening agent, application duration, chemical additives, and remineralizing agents (Alkahtani et al., 2020).

Negative effects of tooth whitening include tooth hypersensitivity (Chen et al., 2021; Epple et al., 2019; Rodríguez‐Martínez et al., 2019; de Sá et al., 2021), and gingival irritation (Carey, 2014), as well as damage of the natural organic matrix of enamel and dentin (Epple et al., 2019), resulting in reduced microhardness (Ferreira et al., 2021; Yang et al., 2021) and surface roughening (de Sá et al., 2021). A potential explanation for the occurrence of bleaching sensitivity has recently been provided (Chen et al., 2021). According to Chen and co‐workers, the penetration of hydrogen peroxide inside the pulp cavity (Bernardi et al., 2021) induces cytotoxicity and pain conduction in dental pulp stem cells via intracellular reactive oxygen species.

In light of these problems associated with current methods of tooth whitening, advanced approaches (Alkahtani et al., 2020) have been developed and tested. These range from the use of lasers (Karanasiou et al., 2021) and ozone (Dietrich et al., 2021) to the application of photosensitizers (Li et al., 2021) and hydrogen peroxide‐containing hydrated calcium silicate (Yang et al., 2021).

Being aware of the ability of boron‐doped diamond (BDD) electrodes to produce various reactive oxygen species during the electrolysis of water, their use might also be an option for developing a well‐controllable bleaching method. Previous work has shown that such electrodes may be applied for disinfecting dental implants (Koch, Burkovski, et al., 2020; Koch, Göltz, et al., 2020) and root surfaces and root canals (Böhm et al., 2019).

Given the similarity of the peroxides produced during electrolysis and commercially available bleaching agents, it was the goal of this in vitro study to compare a standard in‐office bleaching system with electrochemical bleaching using BDD electrodes

## 2 MATERIALS AND METHODS

**Figure 1:**
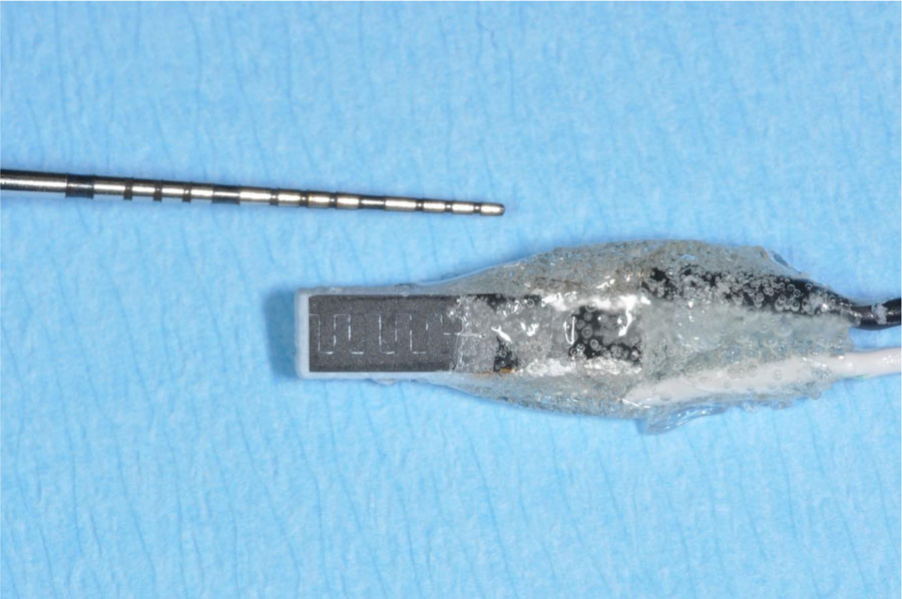
Boron‐doped diamond electrode was used in this study. A ceramic plate was coated with boron‐doped diamond, subsequently separating anode and cathode using a laser. The fabrication process and the electrode application for disinfecting dental implant surfaces have been described previously (Böhm et al., 2019; Koch, Burkovski, et al., 2020; Koch, Göltz, et al., 2020)

Following extraction, teeth were stored in formalin and roots were removed by horizontal cutting using a diamond band saw (EXAKT 300; EXAKT Advanced Technologies GmbH, Norderstedt, Germany). The crowns were not cleaned and split into two groups (*n* = 10) by an independent person not familiar with the study design. In the control group (OPAL), a chemically activated in‐office bleaching system based on 40% hydrogen peroxide (Opalescence Boost; Ultradent Products, Cologne, Germany) was applied for 20 min. In the test group (BDD), specimens were immersed in 1.5 ml 0.67 M NaCl solution and covered before starting electrolysis with a BDD electrode (Figure 1) for 20 min. The electrode was attached to a laboratory power supply set at 5.5–7.5 V and 50 mA.

Before the bleaching procedure, visual shade selection (Czigola et al., 2021) was done by an experienced clinician using the VITA classical A1–D4 shade guide (VITA Zahnfabrik H. Rauter, Bad Säckingen, Germany). Using a white backdrop, standardized photographs of the crowns were taken at a distance of 30 cm before and after bleaching with the previously selected shade depicted as reference (Klinke et al., 2021; Preethi Suganya et al., 2020). The digital camera (Nikon D90; Nikon Europe, Amsterdam, Netherlands) equipped with an objective lens (AF‐S VR Micro‐NIKKOR 105 mm; Nikon Europe) was set at 125/F40 and the flash (Speedlight SB‐29s; Nikon Europe) was set at M1/4.

**Figure 2:**
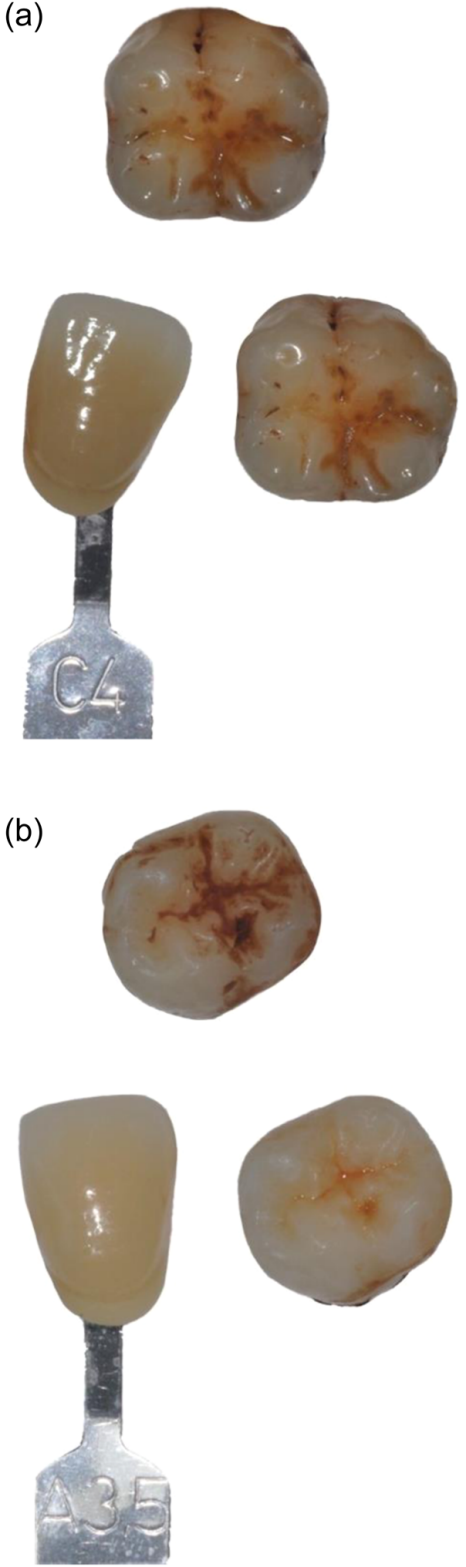
Representative samples of teeth bleached with boron‐doped diamond electrodes (a) and Opalescence (b), respectively. In all cases, pictures were shown as a combination of before and after bleaching as well as with the shade selected before bleaching.

The pictures were shown as a combination of before and after bleaching as well as with the shade selected before bleaching (Figure 2). The photographs were presented to the examiners on a television screen in three series, each with a randomized order of samples. Judgment was done during a single session with each examiner who was asked to judge the bleaching result as 0 (no bleaching), 1 (low bleaching effect), 2 (medium bleaching effect), and 3 (high bleaching effect). The single raters represented different levels of experience (groups) and consisted of final year dental students, dental assistants, and dentists with three representatives in each group.

Statistical analysis included intrarater agreement of single raters and intrarater agreement within groups applying Gwet's AC2 coefficient with quadratic weights (Gwet, 2014). Comparisons between the groups of raters as well as comparisons between the bleaching methods were based on Shapiro–Wilk tests and two‐way analysis of variance of aligned rank transformed data (Wobbrock et al., 2011). All calculations were done using the R software package (R, The R Foundation for Statistical Computing, Vienna, Austria; www.R-project.org) with the level of significance set at *α* = .05.

## 3 RESULTS

The mean rating values in each group of judges ranged from 2.700 to 3.600 for samples bleached using BDD electrodes while values between 4.333 and 4.900 were recorded following in‐office bleaching (Table 1).

**Table 1:** Mean values and standard deviations of summed up ratings recorded for the two different bleaching methods.

Group	BDD		OPAL	
	Mean	SD	Mean	SD
Student	3.600	2.634	4.900	2.746
Dentist	2.833	2.465	4.333	2.644
Assistant	2.700	2.200	4.500	2.910

The ratings provided by the single judges showed levels of reliability ranging from fair to substantial according to the Landis–Koch scale (Table 2a). In the group of dentists, the ratings provided by two individuals did not significantly differ from random scoring (*p* = .1830 and *p* = .1312). Considering the three groups of raters (Table 2b), the level of reliability ranged from fair to moderate, with both dentists' and assistants' ratings not significantly differing from random scoring (*p* = .1830 and *p* = .1312).

**Table 2a:** Intrarater reliability of single raters based on three judgments of 20 items.

Group	Rater	Coefficient	Standard error	*p* value	Landis–Koch
Student	1	0.4098	0.0919	.0468	Moderate
	2	0.6077	0.0367	.0036	Substantial
	3	0.2161	0.0449	.0406	Fair
Dentist	1	0.6272	0.0983	.0237	Substantial
	2	0.2176	0.1086	.1830	Fair
	3	0.2020	0.0814	.1312	Fair
Assistant	1	0.5023	0.0569	.0126	Moderate
	2	0.4087	0.1102	.0656	Moderate
	3	0.2671	0.0370	.0186	Fair

*Note*: Landis–Koch scale for interpreting Gwet's AC2 coefficients: (0.8–1) = *Almost Perfect*; (0.6–0.8) = *Substantial*; (0.4–0.6) = *Moderate*; (0.2–0.4) = *Fair*; (0–0.2) = *Slight*; (−1 to 0) = *Poor*. The *p* value refers to the test against the null hypothesis of purely random scoring.

**Table 2b:** Intrarater reliability within groups based on sums of three judgments of 20 items.

Group	Coefficient	Standard error	*p* value	Landis–Koch
Student	0.4191	0.0937	.0465	Moderate
Dentist	0.2418	0.2211	.3882	Fair
Assistant	0.3704	0.1796	.1753	Fair

*Note*: Landis–Koch scale for interpreting Gwet's AC2 coefficients: (0.8–1) = *Almost Perfect*; (0.6–0.8) = *Substantial*; (0.4–0.6) = *Moderate*; (0.2–0.4) = *Fair*; (0–0.2) = *Slight*; (−1 to 0) = *Poor*. The *p* value refers to the test against the null hypothesis of purely random scoring.

The Shapiro–Wilk test indicated a nonnormal distribution of measurement values (*p* < .0001). Consequently, analysis of variance of aligned rank transformed data was applied using group (Student, Dentist, Assistant) and method (BDD, OPAL) as fixed factors, while rating constituted the variable in the model (Table 3). Neither the factor group nor the interaction group method had a significant effect on the ratings, whereas the bleaching method had a significant effect on ratings (*p* = .0005).

**Table 3:** Analysis of variance of aligned rank transformed data with factors group (Student, Dentist, Assistant) and method (BDD, OPAL).

Factor	Degree of freedom	Residual degree of freedom	*F* value	*p* value
Group	2	174	1.39734	.2500
Method	1	174	12.52712	**.0005**
Group:method	2	174	0.13641	.8726

*Note*: The ratings recorded were seen as variables. Significant differences (*p* < .05) are shown in bold.

## 4 DISCUSSION

This pilot in vitro study was aimed at evaluating the possibility of using BDD electrodes for in‐office tooth whitening procedures. While the technology is still far from clinical application, a handheld device may be envisaged, which allows for the selective whitening of single teeth using applicators similar to current caries infiltration devices.

A bleaching effect of BDD electrodes as used in this study was present, but was significantly lower as compared to a commercially available, chemically activated in‐office bleaching system. In both treatment groups, specimens were exposed to the bleaching agent only once for 20 min, while in clinical situations, multiple treatment cycles would be applicable (Youssef, 2021). It may therefore be argued that the concentration of reactive oxygen species responsible for the whitening effect was lower in the group using BDD electrodes (Maran et al., 2020).

The primary goal of this study was to evaluate the potential of BDD electrodes for tooth whitening in a simplistic in vitro setup, resulting in several limitations that had to be accepted. The raters recruited for this study obviously acted on different scales reaching only low levels of intrarater agreement on a personal as well as on a group level. Despite that, all raters unanimously judged BDD electrodes as being less effective in bleaching as compared to commercial in‐office products.

Besides the in vitro (Kwon et al., 2020) nature of this pilot study, several limitations have to be kept in mind when interpreting the findings of this experiment. The ultimate test for a novel tooth whitening method would of course be to conduct a clinical study that, however, is not possible in the current status due to regulatory reasons. Teeth were not mechanically cleaned before bleaching as would be done in a clinical situation applying dentifrice (Ortega‐Moncayo et al., 2021) or enamel microabrasion (Bernardi et al., 2021). In addition, specimens were randomly allocated to the two study groups without performing any matching with respect to tooth shade and tooth type.

The shade selection method applied here may be seen ambiguously as it has been described not to lead to consistent results (Czigola et al., 2021). In a clinical situation, the surrounding of a tooth, that is, hard and soft tissues affect the appearance of a specific tooth and subjective judgment is also affected by various factors such as skin color, lighting conditions (Revilla‐León et al., 2021), and viewing distance (Klinke et al., 2021). For standardization purposes, it appeared to be best to use a uniform white backdrop for taking pictures, which were repeatedly presented under standardized lighting conditions. A confounder may be seen in the fact that teeth were shown at high magnification as compared to a true‐scale clinical situation.

The use of a digital spectrophotometer (e.g., VITA Easyshade V; VITA Zahnfabrik H. Rauter, Bad Säckingen, Germany) or an intraoral scanning device (TRIOS 4; 3Shape A/S, Copenhagen, Denmark) would have been an alternative for objective shade determination (Preethi Suganya et al., 2020; Revilla‐León et al., 2021; Tabatabaian et al., 2021; Youssef, 2021). While these advanced shade selection methods unanimously are described as being more precise, the clinical relevance of such measurements has been questioned in a recent meta‐analysis as these may be too sensitive and differences recorded with such instruments may not be detectable by the unaided human eye (da Rosa et al., 2020).

## AUTHOR CONTRIBUTIONS

*Conceptualization*: Andreas Burkovski and Matthias Karl. *Methodology*: Victor Palarie. Investigation: Virgilia Klär and Tanja Grobecker‐Karl. *Writing—original draft preparation*: Matthias Karl and Victor Palarie. *Writing—review and editing*: Virgilia Klär and Tanja Grobecker‐Karl. *Visualization*: Andreas Burkovski. *Supervision*: Andreas Burkovski. *Project administration*: Victor Palarie and Andreas Burkovski.

## CONFLICT OF INTEREST

The author Matthias Karl reports a conflict of interest as the inventor of the boron‐doped diamond electrodes used here.

## Data Availability

Research data are not shared.
